# Rapid community "Healthy Heart" screening to identify people at high risk of cardiovascular events

**DOI:** 10.1371/journal.pone.0358999

**Published:** 2026-09-29

**Authors:** Kiran Roest, Catherine Kelly, Tracy Marshall, Emily Whales, Lisa Taylor, Rachel Hatfield, Kate Bunyan, Luke Dawson, Steve Roest, Julia Newton

**Affiliations:** 1 PocDoc, Vital Signs Solutions Ltd, Cambridge, United Kingdom; 2 Health Innovation North East and North Cumbria, Newcastle upon Tyne, United Kingdom; 3 Howbeck Healthcare, Cheshire, United Kingdom; University of Porto, Faculty of Medicine, PORTUGAL

## Abstract

**Objectives:**

To evaluate the proof-of-concept feasibility of identifying individuals at high risk of cardiovascular disease through community-based PocDoc Healthy Heart point-of-care screening in a large, ethnically diverse, disadvantaged real-world cohort in the United Kingdom.

**Methods:**

Proof-of-concept, non-randomised, non-interventional real-world cardiovascular disease risk case finding study in adults aged 18 years or older in real-world communities across the United Kingdom. Main outcome measures were identification of individuals with high risk of cardiovascular disease, defined as QRISK3 score ≥10%; and individuals meeting the criteria for weight loss, smoking cessation, or hypertension management services.

**Results:**

A total of 4,256 individuals (mean age 49 years, 57% male, 31% body mass index ≥30 kg/m^2^) were included in the Primary Cohort. The Targeted Outreach and Workplace sub-cohorts comprised 1,970 and 756 individuals, respectively. In the Primary Cohort, 35% (1,486/4,256) had high (>5.0 mmol/L) total serum cholesterol, 10% were smokers, 10% met criteria for weight loss services and 19% met criteria for hypertension management services. QRISK3 scores were generated for individuals who had not previously had a cardiovascular disease event (~90% of screened individuals). A QRISK3 score ≥10% was found in 20% (784/3,842) of individuals in the Primary Cohort, 26% (457/1,743) in the Targeted Outreach sub-cohort, and 6% (41/701) in the Workplace sub-cohort. In the Primary Cohort, 60% (472/784) of individuals with a QRISK3 score ≥10% were not being treated with a lipid-lowering therapy at the time of testing.

**Conclusions:**

In this proof-of-concept study, community-based point-of-care screening identified individuals with elevated cardiovascular risk and unmet cardiovascular risk management needs. These findings are hypothesis-generating and provide the rationale for further controlled studies with systematic follow-up and health-economic analyses to evaluate whether this approach can deliver improved clinical and system-level outcomes.

**Trial registration:**

ClinicalTrials.gov NCT06258005

## Introduction

Although cardiovascular disease (CVD) is a leading cause of death globally, most premature deaths caused by CVD are preventable by risk factor modification [1]. Dyslipidaemia, obesity, hypertension and diabetes are key modifiable risk factors for CVD and are targeted by preventative medicine initiatives such as the NHS Health Check (NHS-HC) in the United Kingdom (UK) [2,3]. The NHS-HC is a mandated national prevention programme in England that offers adults aged 40–74 years without pre-existing vascular disease a cardiovascular risk assessment every five years. The programme is delivered primarily through general practice and includes measurement of blood pressure (BP), lipid levels, body mass index (BMI), physical activity, alcohol consumption, smoking status, and family history, with QRISK2 or QRISK3 assessment to calculate a 10-year CVD risk. Individuals identified as high risk (≥10% 10-year CVD risk) are offered interventions including lifestyle advice, lipid-lowering therapy, and referral to specialist services. However, the NHS-HC programme currently only reaches a minority of those eligible for the programme and struggles to reach people in deprived parts of the UK, where the burden of CVD and comorbid conditions is the highest [4,5].

Barriers to NHS-HC CVD risk screening in the general population in England include lack of awareness or knowledge, competing priorities, including work commitments, and difficulty obtaining an appointment with a general practitioner (GP) [6]. Furthermore, because of pressures on primary care, some GP surgeries are not prioritising preventative healthcare compared to acute care appointments. Consequently, only 27% of eligible patients have had an NHS-HC in the past 5 years and uptake may be as low as 10% in some areas [7]. Targeted community and workplace screening initiatives may overcome some of these barriers, and identify individuals at high risk of CVD who are unaware of their risk [8].

Risk-factor modification interventions such as smoking cessation, weight loss management, diabetes and hypertension management, and lipid lowering therapy, are key strategies for reducing CVD risk among high-risk individuals [9]. Costs and the requirement for specialised equipment have limited the large-scale implementation of community CVD risk assessment, particularly where point-of-care lipid testing, an important component of CVD risk assessment, is included [10,11].

The PocDoc Healthy Heart Screen includes a digitally integrated point-of-care lipid-testing microfluidic device embedded within a cardiovascular screening application that uses the QRISK3 risk-prediction algorithm [12]. QRISK3 uses a combination of cardiovascular risk factors to estimate the risk of a CVD event within a ten year period. The UK’s NHS defines high risk as a QRISK3 score ≥10% [13]. This study evaluated the feasibility the PocDoc Healthy Heart device to identify individuals at high risk of CVD through point-of-care screening in a large, ethnically diverse, socioeconomically disadvantaged real-world cohort in the United Kingdom.

This proof-of-concept evaluation assessed whether community-based point-of-care screening could identify high-risk individuals outside traditional primary care pathways. It used a pragmatic, observational design that prioritised real-world feasibility over controlled experimental conditions. The findings should therefore be interpreted as hypothesis-generating rather than as an evaluation of comparative performance or system-level impact.

## Methods

The primary aim of this community screening study (NCT06258005) was to identify individuals with a high risk of CVD, defined as having a QRISK3 score ≥10%, the threshold at which NICE Guideline NG238 recommends consideration of CVD risk reduction interventions following an informed discussion [9]. Additional aims included the estimation of proportions of screened individuals that could be eligible for referral to other NHS risk reduction programmes, regardless of QRISK3 score, including weight loss management, smoking cessation and hypertension management. Eligibility criteria of BMI > 30 kg/m^2^ for White and >27 kg/m^2^ for Asian/Black/ethnic minority people; and a diagnosis of diabetes, hypertension or both was used for weight loss management [14]. Smoking cessation eligibility criteria included any level of current tobacco smoking, and hypertension management eligibility criteria were BP reading recorded as 140/90 mm Hg or higher or 150/90 mm Hg or higher if aged >80 years (after repeat readings) [15].

Individuals from across the United Kingdom were included in the study (S1 Fig). Participants had access to approved mobile devices to run a PocDoc mobile application (app) either via private ownership or via community screening partners. Lifestyle and dietary recommendations, and referral to consult their GP were offered based on the PocDoc Healthy Heart screening results, with participants with one or more risk factors being offered advice (S1 Fig).

### Study cohorts

The Primary Cohort included all participants who successfully completed the PocDoc screening procedure. Two sub-cohorts were derived from the Primary Cohort: a Targeted Outreach Sub-Cohort, which included participants screened in community settings identified as being in areas with high levels of undiagnosed CVD risk; and a Workplace Sub-Cohort, which included participants screened at workplaces. Participants screened outside the Targeted Outreach and Workplace cohorts were screened in pharmacy or home settings. Screening locations were selected in collaboration with local health authorities (Health Innovation North-East and North Cumbria) and community partners (Howbeck Healthcare) to target areas with high levels of socioeconomic deprivation, low NHS-HC uptake, and ethnically diverse populations known to have elevated cardiovascular risk. Individual-level socioeconomic status data was not systematically collected. However, screening locations were purposefully selected in areas of high socioeconomic deprivation, particularly for the Targeted Outreach Sub-Cohort, to address health inequalities in cardiovascular screening access. Targeted Outreach Sub-Cohort screening sites included shopping centres, leisure centres, places of worship (churches, mosques), football stadiums, women’s groups, foodbanks, asylum seeker forums, farmers’ markets, and Traveller events – venues with high footfall of individuals likely to face barriers to traditional primary care access. Workplace Sub-Cohort screening was conducted at employer sites that agreed to offer screening to employees during working hours. Home screening was offered to participants who self-referred through the PocDoc platform in partnership with local pharmacies. Recruitment was passive, with participants voluntarily approaching screening stations at community events or requesting home testing. No formal advertising campaigns or targeted invitations were used. Healthcare professionals staffing screening events provided information about the screening programme and obtained consent from all participants.

### Screening procedure

Screening using the PocDoc Healthy Heart device was conducted between June 23, 2023 and June 30, 2024 as described elsewhere and summarised in S2 and S3 Figs [12]. The digitally integrated PocDoc Healthy Heart test comprises a single-use microfluidic PocDoc lipid-panel assay, measurements of height, weight, and BP, and a medical questionnaire. The analytical performance of the PocDoc lipid panel has been previously validated against standard laboratory methods [12]. The device received regulatory clearance as a medical device under UK regulations (UKCA marking) and operates within the quality standards required for point-of-care testing in the UK. The Healthy Heart device includes a smartphone application that runs a full QRISK3 assessment using the acquired data. QRISK is used routinely in clinical practice to determine the risk of an individual suffering a cardiovascular event over the next decade [13,16]. PocDoc is the largest provider of QRISK3 scores in the UK outside of the NHS. For this study, a QRISK3 score ≥10% was treated as meeting the threshold for consideration of CVD risk-reduction interventions under NICE Guideline NG238. Separate prespecified criteria were used to identify participants for whom hypertension-management, weight-management, or smoking-cessation services could be considered [17,18].

BP measurement was an optional component of the screening protocol. Not all participants completed all screening components due to the pragmatic nature of the community-based testing, time constraints at screening events, equipment availability, or participant preference. QRISK3 scores could only be calculated for participants without prior CVD events and with sufficient data to complete the algorithm. The proportion of participants completing each screening component is reported in the Results section.

### Ethics

The study was conducted in accordance with the Declaration of Helsinki, and all participants provided written informed consent. Ethics approval was granted under the Integrated Research Application System (IRAS number 325988).

### Patient and public involvement

Patients and members of the public were not involved in the design, conduct, reporting, or dissemination of this research.

### Statistical analysis

All analyses were descriptive. Categorical data are presented as counts and percentages, with the relevant analysis population as the denominator. Continuous data are summarized as the number of observations (n), mean and standard deviation (SD).

### Measures to mitigate potential bias

Several authors (KR, KB, LD, SR) are employees and shareholders of the company developing the PocDoc device and application. To mitigate potential bias, the study was designed and conducted in collaboration with independent NHS organizations (Health Innovation North-East and North Cumbria, Howbeck Healthcare) who were involved in site selection, participant recruitment, and data collection. The study protocol was prospectively registered (NCT06258005) prior to data collection. Ethics approval was obtained from an independent NHS Research Ethics Committee (IRAS 325988). Raw data collection was performed by healthcare professionals employed by NHS partner organizations, not by company employees. The statistical analysis followed a pre-specified plan using only descriptive methods appropriate for the observational study design, thus minimizing opportunities for selective reporting or data manipulation. Notwithstanding these measures, the involvement of commercial co-authors in manuscript preparation represents an inherent limitation that readers should consider when interpreting the findings.

## Results

### Screening cohorts

The total number of participants approached or the denominator population eligible for screening was not systematically collected across screening venues, limiting our ability to calculate formal uptake rates. This absence of uptake denominators means that observed proportions of high-risk participants reflect the characteristics of those who self-selected to participate and cannot be generalised to the wider populations of the screening locations. Participation was entirely voluntary, with participants self-selecting to attend community screening events. The characteristics of those who participated (Table 1) may differ from non-participants in unmeasured ways. The Targeted Outreach Sub-Cohort included high representation of ethnic minorities (47% Non-White vs. 26% in the Primary Cohort) and high rates of previous CVD events (6% vs. 2% in the Workplace Sub-Cohort), suggesting engagement with high-risk communities. However, we cannot determine whether those who declined screening or did not attend events differ systematically in cardiovascular risk profile from those who participated.

**Table 1:** Baseline and clinical characteristics.

	Primary cohort N = 4256	Targeted Outreach sub-cohort N = 1970	Workplace sub-cohort N = 756
Sex			
Male, n (%)	2413 (57%)	810 (41%)	369 (49%)
Female, n (%)	1843 (43%)	1160 (59%)	387 (51%)
Race			
White, n (%)	3145 (74%)	1049 (53%)	697 (92%)
South Asian, n (%)	489 (11%)	414 (21%)	22 (3%)
Black African/Caribbean, n (%)	245 (6%)	219 (11%)	9 (1%)
Other, n (%)	352 (8%)	269 (14%)	27 (4%)
Not reported/Missing, n (%)	25 (1%)	19 (1%)	1 (0%)
Age, mean years (SD)	49 (14)	49 (14)	43 (11)
< 40 years, n (%)	966 (23%)	525 (27%)	290 (38%)
≥ 40 years to <75 years, n (%)	3138 (74%)	1324 (67%)	466 (62%)
≥ 75 years, n (%)	150 (4%)	120 (6%)	0 (0%)
BMI, kg/m^2^, mean (SD)	28.2 (5.7)	28.7 (5.6)	27.9 (5.9)
< 25 kg/m^2^, n (%)	1,244 (29%)	515 (26%)	239 (32%)
≥ 25 to <30 kg/m^2^, n (%)	1,714 (40%)	775 (39%)	301 (40%)
≥ 30 to <35 kg/m^2^, n (%)	830 (20%)	431 (22%)	155 (21%)
≥ 35 kg/m^2^, n (%)	468 (11%)	249 (13%)	61 (8%)
Health Check including cholesterol test in past 5 years?			
Yes, n (%)	1,220 (29%)	606 (31%)	225 (30%)
No, n (%)	1,923 (45%)	1,114 (57%)	401 (53%)
No response (%)	1,113 (26%)	250 (13%)	130 (17%)
Prior CVD event (i.e., secondary prevention patient)*, n (%)	220 (5%)	119 (6%)	13 (2%)
Smoking status, n (%)			
Non-smoker	3,096 (73%)	1,439 (73%)	608 (80%)
Ex-smoker	748 (18%)	287 (15%)	111 (15%)
Light smoker (<10 cigarettes/day)	216 (5%)	128 (6%)	22 (3%)
Moderate smoker (10–19 cigarettes/day)	142 (3%)	78 (4%)	10 (1%)
Heavy smoker (≥20 cigarettes/day)	51 (1%)	38 (2%)	2 (0%)
No response	3	0	3
Diabetes status, n (%)			
Type 1 diabetes	24 (1%)	11 (1%)	3 (0%)
Type 2 diabetes	231 (5%)	145 (7%)	10 (1%)
Angina or MI in 1st degree relative younger than 60 years, n (%)	795 (19%)	378 (19%)	103 (14%)
Chronic kidney disease, stage 3–5	44 (1%)	25 (1%)	4 (1%)
Atrial fibrillation, n (%)	111 (3%)	47 (2%)	10 (1%)
Antihypertensive therapy, n (%)	706 (17%)	313 (16%)	60 (8%)
Migraine, n (%)	693 (16%)	310 (16%)	115 (15%)
Rheumatoid arthritis, n (%)	213 (5%)	120 (6%)	15 (2%)
Systemic lupus erythematosus, n (%)	27 (1%)	10 (1%)	4 (1%)
Severe mental illness, n (%)**	264 (6%)	155 (8%)	15 (2%)
Atypical antipsychotic medication, n (%)	67 (2%)	38 (2%)	10 (1%)
Regular oral glucocorticoid therapy, n (%)	157 (4%)	57 (3%)	19 (3%)
Erectile dysfunction (diagnosis or treatment), n (%)	82 (2%)	22 (1%)	7 (1%)

*Secondary prevention patients are defined as patients who have experienced a prior CVD event and who have been entered onto the CVD register.

**Schizophrenia, bipolar disorder, moderate/severe depression.

BMI, body mass index; CVD, cardiovascular disease; MI, myocardial infarction; SD, standard deviation.

Because of the community nature of the screening, equipment limitations and individual’s preferences, some data fields were not collected for some participants. BP measurements were taken for only 63% of participants in the Primary Cohort. Lipid levels were measured successfully for all participants. Sufficient data was obtained for QRISK3 measurement in 90% of participants. These missing data mean that estimates of hypertension prevalence and cardiovascular risk are based on incomplete samples and may not be representative of the full screened population.

Participants in the Primary Cohort (N = 4,256) had a mean age of 49 years; were 57% male; and most were overweight or obese. Only 29% of the Primary Cohort participants stated they had had a health check including a lipid panel in the past five years, 45% reported they had not, and 26% did not answer the question (Table 1). Compared with the Workplace Sub-Cohort (N = 756), the Targeted Outreach Sub-Cohort (N = 1,970) was more ethnically diverse, older, had higher BMI, had had fewer prior cholesterol checks, had more prevalent history of CVD and had a higher proportion of current smokers (Table 1). These baseline differences between sub-cohorts preclude any meaningful direct comparison of QRISK3 or risk factor rates across cohorts.

### Identification of participants with high total cholesterol, HDL cholesterol, non-HDL cholesterol and triglycerides

Serum total cholesterol, HDL cholesterol, non-HDL cholesterol, and triglycerides were quantified in 4,256 participants in the Primary Cohort, in 1,970 in the Targeted Outreach Sub-Cohort and in 756 in the Workplace Sub-Cohort (Table 2). In the Primary Cohort, 35% had total serum cholesterol levels ≥5 mmol/L; 21% had serum non-HDL ≥ 4 mmol/L; 25% had HDL ≤ 1.0 mmol/L for males/ ≤ 1.2 mmol/L for females; 27% had triglycerides ≥2.3 mmol/L; and 20% had a triglyceride:HDL ratio ≥4 (Fig 1). Similar patterns were observed in the Targeted Outreach and Workplace Sub-Cohorts, though these descriptive observations should be interpreted considering the baseline differences between cohorts noted above.

**Table 2:** Serum lipid concentrations in the Primary cohort and Targeted Outreach and Workplace cohorts.

	Primary cohort N = 4,256	Targeted Outreach sub-cohort N = 1,970	Workplace sub-cohort N = 756
Total cholesterol (mmol/L), mean (SD)	4.5 (1.3)	4.7 (1.2)	4.6 (1.2)
HDL (mmol/L), mean (SD)	1.4 (0.4)	1.5 (0.4)	1.4 (0.4)
Non-HDL (mmol/L), mean (SD)	3.3 (0.8)	3.4 (0.8)	3.4 (0.9)
Total cholesterol/HDL, mean (SD)	3.3 (0.9)	3.4 (0.9)	3.5 (0.9)
Triglycerides (mmol/L), mean (SD)	1.8 (0.6)	1.8 (0.6)	1.7 (0.6)

NHS risk threshold cutoff values are defined as total cholesterol ≥5 mmol/L; non-HDL ≥ 4 mmol/L; HDL in males: ≤ 1.0 mmol/L; HDL in females: ≤ 1.2 mmol/L; triglycerides ≥2.3 mmol/L; triglyceride:HDL ratio ≥4.

HDL, high-density lipoprotein; NHS, National Health Service; SD, standard deviation.

**Fig 1:**
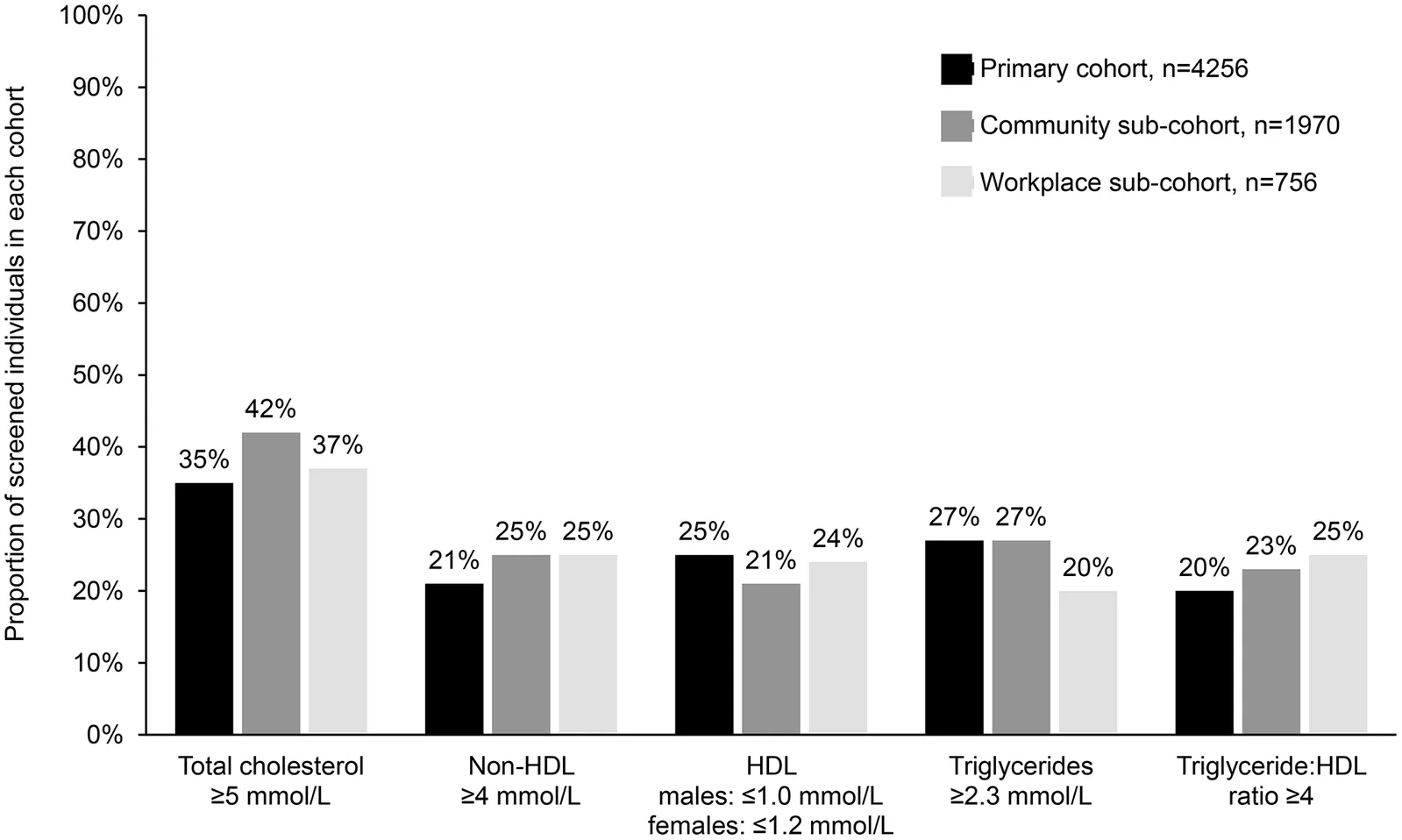
Proportion of screened individuals with cholesterol or triglyceride values exceeding NHS risk threshold cutoff values. NHS risk threshold cutoff values are defined as total cholesterol ≥5 mmol/L; non-HDL ≥ 4 mmol/L; HDL in males: ≤ 1.0 mmol/L; HDL in females: ≤ 1.2 mmol/L; triglycerides ≥2.3 mmol/L; triglyceride:HDL ratio ≥4. HDL, high-density lipoprotein; NHS, National Health Service.

### Identification of participants with potential hypertension

At screening, 3,550/4,256 (83%) participants stated that they were not taking any antihypertensive medication, and 706 (17%) stated that they were on antihypertensive treatment. BP data from 2,671/4,256 (63%) participants in the Primary Cohort were available for inclusion in the CVD risk assessment. As BP data were missing for 37% of the Primary Cohort, the following estimates are based on those who provided BP readings and may not be representative of the full screened cohort.

A total of 810/2,671 (30%) had BP readings above the normal range (i.e., systolic BP > 140 mm Hg or diastolic BP > 90 mm Hg if aged 80 years or younger; 150/90 or higher if aged >80 years), including 610/2,671 (23%) that were not on any antihypertensive treatment. Additionally, among the 474 participants that were on antihypertensive treatment and that provided their BP, 210/474 (44%) had BP above the recommended range.

### Identification of participants meeting prespecified criteria for consideration of existing preventative care programmes

QRISK3 scores were generated for participants who had not previously had a CVD event, which was approximately 90% of all screened participants (Table 3). A total of 20% (784/3,842) of participants in the Primary Cohort, 26% (457/1,743) in the Targeted Outreach Sub-Cohort, and 6% (41/701) in the Workplace Sub-Cohort had a QRISK3 score ≥10% (i.e., a ≥ 10% or greater risk of developing CVD within 10 years) making them eligible for consideration for CVD risk reduction in line with NICE Guideline 238 (Table 3) [9]. The observed variation in QRISK3 scores ≥10% between cohorts (26% in Targeted Outreach vs. 20% in Primary vs. 6% in Workplace) reflects differences in the underlying characteristics of populations accessing these different screening settings (Table 1), rather than representing a controlled comparison of screening effectiveness.

**Table 3:** Identification of screened participants meeting criteria for consideration of CVD risk reduction, weight management or smoking cessation services.

	Primary cohort N = 4,256	Targeted Outreach sub-cohort N = 1,970	Workplace sub-cohort N = 756
Eligibility for CVD risk reduction referral by QRISK3 score			
Screened participants with QRISK3 score, n/N (%)	3,842/4,256 (90)	1,743/1,970 (88)	701/756 (93)
Proportion of screened participants with QRISK3 score ≥10%, n/n (%)	784/3,842 (20)	457/1,743 (26)	41/701 (6)
Screened participants with QRISK3 score ≥10% by BMI subgroup, n/n (%)			
BMI < 25 kg/m^2^	187/784 (24)	102/457 (22)	8/41 (20)
BMI ≥ 25 to <30 kg/m^2^	338/784 (43)	190/457 (42)	14/41 (34)
BMI ≥ 30 to <35 kg/m^2^	162/784 (21)	94/457 (21)	17/41 (41)
BMI ≥ 35 kg/m^2^	97/784 (12)	71/457 (16)	2/41 (5)
Use of lipid-lowering therapy in participants with QRISK3 score ≥10%, n/n (%)			
On statin therapy	308/784 (39)	174/457 (38)	14/41 (34)
Not on statin therapy	472/784 (60)	282/457 (62)	27/41 (66)
Missing	4/784 (1)	1/457 (<1)	0
Eligibility for referral for smoking cessation, n/n (%)*	409/4,256 (10)	244/1,970 (12)	34/756 (4)
Eligibility for referral for weight loss management, n/n (%)**	407/4,256 (10)	231/1,970 (12)	31/756 (4)
Eligibility for hypertension management, n/n (%)^†^	810/4,256 (19)	514/1,970 (26)	165/756 (22)
Participants given lifestyle or dietary recommendations by HCP	1,714/2,283 (75)	1,Figure290/1,546 (83)	207/419 (49)
Participants recommended to consult GP	760/2,283 (33)	554/1,546 (36)	97/419 (23)

*Any level of current smoking.

**BMI > 30 kg/m^2^ for White and >27 kg/m^2^ for Asian, Black, or other ethnic minorities, together with a diagnosis of diabetes, hypertension or both.

†BP reading recorded as 140/90 or higher or 150/90 or higher if aged >80. BP was unavailable for 37% of the Primary Cohort; the reported proportions therefore reflect incomplete ascertainment and are not prevalence estimates.

BMI, body mass index; BP, blood pressure, CVD, cardiovascular disease; GP, general practitioner; HCP, healthcare professional; n, number.

The proportion of screened participants meeting criteria for consideration for smoking cessation or weight loss management services was 10% across the Primary Cohort whilst 19% of all screened participants were eligible to be considered for hypertension management (Table 3).

In the Primary Cohort, 60% (472/784) of the screened participants with a QRISK3 score ≥10% were not being treated with a lipid-lowering therapy such as a statin. Only 44% of these participants at high-risk of CVD events had previously had a health check including a full lipid panel.

### Referral of participants to onward care pathways

The PocDoc Healthy Heart questionnaire included an optional field that asked whether lifestyle or dietary advice was provided to the person being screened, or whether the person was recommended to visit their GP for follow-up care. A total of 1,714 (75%) of participants answering the question were given lifestyle and/or dietary advice following their PocDoc screen, and 760 (33%) of participants were recommended to visit their GP for follow-up care (Table 3). Whether participants acted on these recommendations was not captured in this study, and referral rates should therefore be interpreted as guidance given rather than confirmed follow-up.

### User experience

Participants in the Primary Cohort and the Targeted Outreach and Workplace Sub-Cohorts reported high likelihoods of recommending the PocDoc test to a family member (Net Promoter Scores of 88, 93 and 90, respectively; S1 Table), and 93% of participants found the PocDoc test more convenient that being tested at a GP surgery (S2 Table). These user experience data reflect self-reported perceptions of acceptability and convenience, and do not constitute evidence of clinical effectiveness or superiority over existing care pathways.

## Discussion

This proof-of-concept study found that community-based point-of-care cardiovascular screening using the PocDoc Healthy Heart device was feasible in the settings studied and can identify participants with elevated CVD risk who had not been previously screened. Over four thousand people participated in the study, with approximately one in five participants having a QRISK score of ≥10% and therefore being at high risk of having a CVD event within the next ten years. The majority of these participants (60%) were not receiving lipid lowering therapy at the time of screening. However, these descriptive findings do not establish clinical benefit or comparative performance against existing screening pathways.

Outreach community screening was conducted in shopping centres, leisure centres, churches, mosques, football stadiums, women’s groups, foodbanks, asylum-seeker forums, farmers’ markets and Traveller events. In this Targeted Outreach Sub-Cohort, which comprised older, more ethnically diverse individuals with high comorbidity levels (Table 1), one in four people for whom a QRISK3 score was calculated had a QRISK3 score of ≥10%. This finding demonstrates that the screening was able to identify people at high risk of CVD. However, it also reflects the characteristics of the self-selected population attending these venues and cannot directly be interpreted as evidence that community screening is more effective than existing pathways, or that it would yield similar rates in other populations or settings. Given the substantial baseline differences between cohorts (Table 1), direct comparisons between screening settings would not be appropriate without statistical adjustment and were not performed in this proof-of-concept study.

Among participants with a QRISK3 score of ≥10%, one in four met the recorded criteria for consideration of hypertension management, six in ten were not receiving lipid-lowering therapy, and eight in ten had a BMI in the overweight or obese range. The study did not assess whether screening recommendations led to treatment initiation, engagement with services, or reduction in CVD risk. These outcomes require prospective evaluation with appropriate comparators and follow-up.

In the Primary Cohort, 813 participants had BP above the prespecified threshold which made them eligible for the hypertension management service; this represented 30% of those with recorded BP and 19% of the full cohort. Among participants who reported antihypertensive treatment and had recorded BP, 44% had a reading above the prespecified treatment target threshold. However, only 63% of the Primary Cohort provided BP readings, and findings may therefore not represent the full screened population. Observations are descriptive and hypothesis-generating; future studies with complete BP data and appropriate controls are needed to determine whether community screening adds value over existing pathways in managing hypertension at the population level. A high proportion of screened participants were eligible for other existing preventative care pathways including weight management and smoking cessation. Although some participants received recommendations to consult their GP, the study did not record whether they attended primary care or engaged with onward services, and no clinical impact can therefore be inferred.

The findings from our study are consistent with previous community reports that community pharmacy and workplace programmes can identify high-risk individuals and improve access to screening outside of primary care [19–30]. The NHS-HC programme has also been delivered in community venues, with pilot studies reporting reach into some underserved groups [31,32]. The present study adds descriptive evidence that integrated point-of-care lipid testing and comprehensive QRISK3 assessment can be delivered across diverse community settings including non-traditional venues such as places of worship, foodbanks, and asylum-seeker forums. In the Primary Cohort, 45% reported no health check in the previous five years, 29% reported such a check and 26% did not answer the question. This pattern suggests that community screening may engage some people without recent lipid testing, but the absence of uptake denominators and self-selected sampling preclude conclusions about population reach and comparative performance. Questions about subsequent adherence, costs and effects on health inequalities also remain [21].

The PocDoc Healthy Heart screen uses a smartphone camera to quantify enzymatic colorimetric reactions from a point-of-care lipid panel in a microfluidic assay, and integrates lipid results alongside other screen-derived measurements including height, weight, BP and medical history to estimate a person’s ten-year CVD risk score (QRISK score) and their healthy heart age [12]. QRISK3 estimates a person’s risk of myocardial infarction (MI) or stroke over a 10-year period and was derived using data from over ten million UK primary care patients [13]. Under NICE Guideline NG238, a risk score of ≥10% is the threshold for offering atorvastatin for primary prevention after an informed discussion of risks and benefits [9].

The implications of community screening for primary care workload requires careful consideration. In this study, 33% of screened participants (760/2,283 responding) were recommended to consult their GP for follow-up care. Acting upon these recommendations could generate additional appointments. However, the study did not record subsequent GP attendance or healthcare utilization and therefore cannot determine the direction or magnitude of any effect on workload.

Early identification of cardiovascular risk could, in principle, facilitate preventive management and potentially reduce acute presentations. However, this study did not assess whether identified risks would have otherwise remained undetected, whether recommended care was initiated or whether acute cardiovascular events were prevented. Any effect on subsequent emergency presentations remains hypothetical.

Electronic transmission of screening results directly to GP records (introduced in the PocDoc application after this study’s conclusion) may reduce reliance on participant-initiated communication but its effect on workflow has not been evaluated here. The balance between prevention workload and averted acute care resource use should be evaluated in future cost-effectiveness and healthcare utilization studies. For the 60% of high-risk participants not on lipid-lowering therapy, initiating statins represents a straightforward, evidence-based intervention that could be delivered efficiently in primary care.

### Limitations of study

This proof-of-concept, real-world study has several limitations. First, missing data for key variables reflects the pragmatic nature of community screening; for example, BP measurements were available for only 63% of the Primary Cohort. Missing data arose from time constraints at community events, equipment availability, participant preference to decline certain measurements, and the optional nature of some screening components. These missing data may introduce selection bias if participants declining measurements differ systematically from those completing the full assessment. Second, potential language barriers may have limited minority group engagement despite targeting ethnically diverse communities. Third, the study relied on participants pursuing referrals to consult their GP if identified as having high CVD risk, with no systematic follow-up to confirm whether referrals were completed. After this study concluded, changes have been made to the PocDoc screening process to enable GPs to receive results directly to overcome this limitation. Fourth, as a proof-of-concept observational study without a control group, we cannot assess the comparative effectiveness of community screening versus standard NHS Health Check delivery, nor can we evaluate long-term clinical outcomes or cost-effectiveness. The absence of systematic data on screening uptake rates represents a limitation in interpreting the reach and potential selection bias of community screening. Those who voluntarily participated in community screening may differ from non-participants in health awareness, health-seeking behaviour, perceived risk, or actual cardiovascular risk burden. This self-selection may lead to either overestimation (if worried participants with symptoms participated preferentially) or underestimation (if healthy participants took part out of curiosity while those with known problems avoided screening) of true community CVD risk prevalence. Future implementation should include systematic capture of uptake rates to better understand reach and representativeness. Finally, the involvement of commercial co-investigators in the study design and manuscript preparation, despite the mitigation measures described, may have influenced study conduct or reporting in ways that are difficult to quantify. Readers should weigh these potential sources of bias when interpreting the findings.

### Priorities for future research

Although individual socio-economic status (SES) data were unavailable for analysis, our targeted approach, i.e., screening in deprived communities, places of worship, foodbanks, and asylum seeker forums, to reach high-risk, underserved groups (Table 1), may support the feasibility of community screening to address access inequalities. Future implementation studies will integrate individual SES measures and longitudinal follow-up to quantify equity impacts using validated metrics (e.g., proportion screened by deprivation quintile, uptake by ethnicity).

Cost-effectiveness analysis was beyond the scope of this proof-of-concept feasibility study and represents an important direction for future research. Preliminary cost considerations suggest that community-based screening may offer potential advantages through reduced primary care facility overhead, batch processing of multiple participants at community events, and reaching populations with low NHS-HC uptake who would otherwise not be screened. However, comprehensive cost-effectiveness evaluation would need to account for equipment costs, healthcare professional time, consumables, referral management, downstream healthcare utilization following identification of high-risk individuals, and most importantly, long-term clinical outcomes including cardiovascular events prevented. Such analyses should be a priority for future studies before widespread implementation can be recommended. A separate economic evaluation of this programme is currently under review and will further address these questions, but detailed cost-effectiveness findings are beyond the scope of the present article.

The NHS-HC initiative is a national risk assessment and management programme that is free at point-of-care for adults aged 40–74 years living in England who do not currently have any vascular disorders and are not being treated for risk factors such as diabetes [33]. However, the uptake of NHS-HC has been poor, with only 27% of eligible participants having had an NHS-HC in the past five years. In some areas this may be as low as 10% [7].

Based on the findings and limitations of this proof-of-concept study, we propose several areas of development for community-based cardiovascular screening programs. First, systematic capture of uptake rates and participation demographics is essential to evaluate reach and identify under-represented groups. Second, direct electronic transmission of results to GP practices should be standard to ensure continuity of care and reduce reliance on patient-initiated follow-up. Third, integration with existing NHS prevention pathways is critical, including pre-arranged referral protocols for weight management, smoking cessation, and hypertension services. Fourth, community screening should target areas with documented low NHS Health Check uptake using local health intelligence to maximize impact on health inequalities. Fifth, standardized training for healthcare professionals delivering community screening ensures consistent quality and appropriate management of results requiring urgent attention. Sixth, partnerships with community organizations (faith groups, community centres, employers) facilitate access to target populations and build trust. Seventh, multi-language materials and culturally competent staff are necessary to maximize engagement with ethnically diverse communities. Finally, formal evaluation frameworks should be embedded from the outset, including long-term follow-up of cardiovascular events, treatment initiation and adherence, cost-effectiveness, and patient-reported outcomes. Such a framework will ultimately support the sustainable delivery of community-based point-of-care testing as a complement to traditional primary care screening.

## Conclusion

This proof-of-concept study found that community-based point-of-care cardiovascular screening using the PocDoc Healthy Heart device was feasible across the real-world settings studied. Individuals with elevated QRISK3 scores and unmet cardiovascular risk management needs were identified through the screens. A substantial proportion of those screened had not previously received a health check, suggesting that community screening may reach individuals with limited prior engagement with preventive services. These findings are descriptive and hypothesis-generating. Controlled studies with uptake denominators, systematic follow-up, appropriate comparators and health-economic evaluation are needed to determine the clinical outcomes, equity of reach and implications for health-service use.

## Supporting information

S1 Fig Map of participating study areas in the United Kingdom. Source: Map data: © OpenStreetMap contributors, ODbL.
(DOCX)

S2 Fig PocDoc testing workflow and clinical referral pathway. BP, blood pressure; GP, general practitioner; HCP, healthcare provider; HDLc, high-density lipoprotein cholesterol; QRISK, Cardiovascular Risk.
(DOCX)

S3 Fig PocDoc point-of-care finger-prick cholesterol test procedure. A) 4-step process involved in the blood cholesterol screening; B) Illustration of the individual components that are assembled together to create the microfluidic assay device; C) The lipid test kit (dimensions 35 mm × 79 mm) requires no electronic components. A companion smartphone application serves as image reader, analyser and results display. HDL, high-density lipoprotein. Original photographs of PocDoc device and assay components. Images are original works created for this publication.
(DOCX)

S1 Table Likelihood of recommending PocDoc test to a family member. Question: “How likely are you to recommend this cholesterol test service to a family member?” Score range: 0 = lowest; 10 = highest. Detractors: individuals scoring 0–6; Passives: individuals scoring 7–8; Promoters: individuals scoring 9–10. Net Promoter Score = % promoters – % detractors.
(DOCX)

S2 Table Perceived convenience of PocDoc test versus test at general practitioner surgery. Question: “How convenient was testing with PocDoc versus going to your GP?” GP, general practitioner.
(DOCX)
