# Supplementary material for: Rapid community "Healthy Heart" screening to identify people at high risk of cardiovascular events
Source: PLoS One. 2026 Sep 29;21(9):e0358999. doi: 10.1371/journal.pone.0358999 (PMC13623158; doi:10.1371/journal.pone.0358999)
Supplement: Supplementary file 1 [file pone.0358999.s001.docx]

## Supplementary figure 1. Map of participating study areas in the United Kingdom.

Source: Map data: © OpenStreetMap contributors, ODbL
