# Supplementary material for: Rapid community "Healthy Heart" screening to identify people at high risk of cardiovascular events
Source: PLoS One. 2026 Sep 29;21(9):e0358999. doi: 10.1371/journal.pone.0358999 (PMC13623158; doi:10.1371/journal.pone.0358999)
Supplement: Supplementary file 2 [file pone.0358999.s002.docx]

## Supplementary figure 2. PocDoc testing workflow and clinical referral pathway

BP, blood pressure; GP, general practitioner; HCP, healthcare provider; HDLc, high-density lipoprotein cholesterol; QRISK, Cardiovascular Risk
