# Supplementary material for: Rapid community "Healthy Heart" screening to identify people at high risk of cardiovascular events
Source: PLoS One. 2026 Sep 29;21(9):e0358999. doi: 10.1371/journal.pone.0358999 (PMC13623158; doi:10.1371/journal.pone.0358999)
Supplement: Supplementary file 3 [file pone.0358999.s003.docx]

## Supplementary figure 3. PocDoc point-of-care finger-prick cholesterol test procedure.

**A)** 4-step process involved in the blood cholesterol screening; **B)** Illustration of the individual components that are assembled together to create the microfluidic assay device; **C)** The lipid test kit (dimensions 35 mm × 79 mm) requires no electronic

components. A companion smartphone application serves as image reader, analyser and results display.

HDL, high-density lipoprotein. Original photographs of PocDoc device and assay components. Images are original works created for this publication.
