# Supplementary material for: Rapid community "Healthy Heart" screening to identify people at high risk of cardiovascular events
Source: PLoS One. 2026 Sep 29;21(9):e0358999. doi: 10.1371/journal.pone.0358999 (PMC13623158; doi:10.1371/journal.pone.0358999)
Supplement: Supplementary file 4 [file pone.0358999.s004.docx]

## Supplementary table 1. Likelihood of recommending PocDoc test to a family member

|  | **Primary cohort (n=2776)** | **Targeted Outreach  sub-cohort (n=1548)** | **Workplace sub-cohort (n=438)** |
| --- | --- | --- | --- |
| Answered, n (%) | 2171 (78) | 1125 (73) | 395 (90) |
| Score, n |  |  |  |
| 0 | 2 | 0 | 0 |
| 1 | 5 | 2 | 0 |
| 2 | 2 | 0 | 0 |
| 3 | 0 | 0 | 0 |
| 4 | 5 | 2 | 0 |
| 5 | 13 | 4 | 2 |
| 6 | 23 | 7 | 3 |
| 7 | 47 | 15 | 8 |
| 8 | 122 | 35 | 21 |
| 9 | 327 | 124 | 74 |
| 10 | 1625 | 936 | 287 |
| Net Promoter Score | 88 | 93 | 90 |

Question: “How likely are you to recommend this cholesterol test service to a family member?”

Score range: 0 = lowest; 10 = highest.

Detractors: individuals scoring 0–6; Passives: individuals scoring 7–8; Promoters: individuals scoring 9–10.

Net Promoter Score = % promoters – % detractors
