# Supplementary material for: Rapid community "Healthy Heart" screening to identify people at high risk of cardiovascular events
Source: PLoS One. 2026 Sep 29;21(9):e0358999. doi: 10.1371/journal.pone.0358999 (PMC13623158; doi:10.1371/journal.pone.0358999)
Supplement: Supplementary file 5 [file pone.0358999.s005.docx]

## Supplementary table 2. Perceived convenience of PocDoc test versus test at general practitioner surgery

|  | **Primary cohort (n=2776)** | **Targeted Outreach  sub-cohort (n=1548)** | **Workplace sub-cohort (n=438)** |
| --- | --- | --- | --- |
| Answered, n (%) | 2315 (83) | 1125 (77) | 395 (96) |
| Less convenient, n (%) | 15 (1) | 8 (1) | 2 (<1) |
| About the same, n (%) | 153 (7) | 66 (6) | 23 (5) |
| More convenient, n (%) | 2147 (93) | 1114 (94) | 397 (94) |

Question: “How convenient was testing with PocDoc versus going to your GP?”

GP, general practitioner
